# Parameter inference in a computational model of haemodynamics in pulmonary hypertension

**DOI:** 10.1098/rsif.2022.0735

**Published:** 2023-03-01

**Authors:** Amanda L. Colunga, Mitchel J. Colebank, REU Program, Mette S. Olufsen

**Affiliations:** ^1^ Department of Mathematics, North Carolina State University, Raleigh, NC, USA; ^2^ University of California, Irvine—Edwards Lifesciences Foundation Cardiovascular Innovation and Research Center, and Department of Biomedical Engineering, University of California, Irvine, CA, USA

**Keywords:** pulmonary hypertension, computational model, parameter inference, cardiovascular modelling

## Abstract

Pulmonary hypertension (PH), defined by a mean pulmonary arterial pressure (mPAP) greater than 20 mmHg, is characterized by increased pulmonary vascular resistance and decreased pulmonary arterial compliance. There are few measurable biomarkers of PH progression, but a conclusive diagnosis of the disease requires invasive right heart catheterization (RHC). Patient-specific cardiovascular systems-level computational models provide a potential non-invasive tool for determining additional indicators of disease severity. Using computational modelling, this study quantifies physiological parameters indicative of disease severity in nine PH patients. The model includes all four heart chambers, the pulmonary and systemic circulations. We consider two sets of calibration data: static (systolic and diastolic values) RHC data and a combination of static and continuous, time-series waveform data. We determine a subset of identifiable parameters for model calibration using sensitivity analyses and multi-start inference and perform posterior uncertainty quantification. Results show that additional waveform data enables accurate calibration of the right atrial reservoir and pump function across the PH cohort. Model outcomes, including stroke work and pulmonary resistance-compliance relations, reflect typical right heart dynamics in PH phenotypes. Lastly, we show that estimated parameters agree with previous, non-modelling studies, supporting this type of analysis in translational PH research.

## 1. Introduction

Patients with a resting mean pulmonary arterial blood pressure (mPAP) greater than 20 mmHg are diagnosed with pulmonary hypertension (PH) [1]. This disease has no cure and, if left untreated, progresses rapidly, leading to thickening and stiffening of the pulmonary vasculature, vascular-ventricular decoupling and right ventricular failure [2,3]. There are five main PH etiologies: pulmonary arterial hypertension (PAH, group 1), PH due to left heart disease (group 2), PH due to lung disease and/or hypoxia (group 3), chronic thromboembolic PH (CTEPH, group 4) and PH with unclear multi-factorial mechanisms (group 5) [4]. Only patients in groups 1 and 4 have PH as their primary disease; in groups 2–5, PH is a comorbidity. Patients with PAH (group 1) and CTEPH (group 4) experience common symptoms early on, including shortness of breath, dizziness, fainting, fatigue, and swelling of the legs and abdomen [5]. Early diagnosis is difficult. Therefore, patients with suspected PH undergo several tests. A definite diagnosis requires invasive pulmonary arterial blood pressure measurements from right heart cardiac catheterization (RHC) [5,6]. PH symptoms do not appear until 1–2 years after disease onset [3]. At this time, patients have typically undergone significant disease progression before diagnosis, limiting and reducing treatment outcomes. Understanding how cardiovascular parameters (e.g. pulmonary vascular resistance (PVR), and compliance) are modulated with the disease can assist in early detection and better therapeutic interventions. We use systems-level computational models with RHC data to study how model parameters and outcomes are modulated with PH.

Mathematical modelling is useful for monitoring and understanding cardiovascular disease progression. Systems-level models with multiple cardiovascular compartments have successfully analysed *in vivo* dynamics [7–9]. For example, Colunga *et al.* [9] used a zero-dimensional systems-level model to predict pressure–volume loops and left ventricular power to understand heart transplant recovery. Kung *et al.* [7] used a similar model to quantify exercise capacity in Fontan patients, an essential indicator of patient survival. The study by Shimizu *et al.* [8] used a zero-dimensional model to study post-operative dynamics in patients with a hypoplastic right ventricle. Their results show that the effectiveness of ventricular repair can be predicted by right ventricular stiffness. These studies used models to predict patient outcomes. As noted by Colunga *et al.* [9], reliable results require that model parameters are identifiable given the model structure and available data. Parameters are identifiable if they influence the model output and can be uniquely determined by available data. A parameter’s influence on model predictions is quantified using local [10,11] and global [12–14] sensitivity analyses. Subset selection algorithms [11,15] determine parameter interdependence and reduce identifiability issues. Schiavazzi *et al.* [16] estimated cardiovascular model parameters by fitting simulations to data from single-ventricle patients with a Norwood physiology. They show that combining local and global identifiability techniques, *a priori*, provides unique and consistent parameter estimates given the available data. Our group [9] used similar methods to analyse data from heart-transplant patients, finding that model predictions align with static RHC data measured at one point and over longitudinal patient recordings.

These previous studies use non-invasive or static data, while others use dynamic time-series data, including pressure waveforms, for model calibration. Marquis *et al.* [17] developed a compartment model of the systemic circulation. This model was calibrated by inferring five identifiable model parameters to simultaneously recorded left ventricular pressure and volume waveforms in rats. Their results showed that estimating these parameters agreed between the dynamic model prediction and the waveform data. The study by Bjørdalsbakke *et al.* [18] compared model sensitivity using static or dynamic outputs from a systemic circulation model. They found that time-averaged global sensitivities of aortic pressure were less influential to systemic resistance than static systolic and diastolic pressure outputs. Gerringer *et al.* [19] used three- and four-element Windkessel models to predict main pulmonary artery pressure waveforms in control and PAH mice. The study matched model simulations to dynamic main pulmonary artery data, showing good agreement with the data. However, the authors did not consider a closed-loop model. These studies demonstrate the importance of employing sensitivity analyses and parameter reduction but they do not discuss what data, static and/or dynamic, are informative for parameter inference. Most clinical protocols only use static data in electronic health records. Though static measurements are extracted from waveform data, storing patient static and dynamic pressure adds complexity to data storage. However, PH time-series pressure data may reveal important markers of disease severity.

The objective of this study is twofold: we investigate (i) how systems-level model calibration is improved by adding dynamic RHC data and (ii) if patient-specific cardiovascular parameters are consistent with the physiological understanding of PH. To do so, we study the impact of model parameters on haemodynamic predictions using local and global sensitivity analyses. To quantify the benefits of adding waveform data in parameter inference, we consider two weighted residual vectors comparing model predictions with static data (systolic, diastolic and mean pressures and cardiac output, CO) and using a combination of static and dynamic data (RHC time-series waveforms). By integrating mathematical modelling, patient-specific data and physiological intuition, we categorize each patient’s functional state, including right atrial, right ventricular, and main pulmonary artery temporal dynamics. In addition, we calculate patient-specific physiological biomarkers, including pressure–volume loops and other markers of PH severity.

## 2. Methods

### 2.1. Ethics and approval

Patient-specific data are obtained from two hospitals, adhering to their respective institutional review board guidelines. De-identified RHC patient data are obtained from the Scottish Pulmonary Vascular Unit at the Golden Jubilee National Hospital, Glasgow, UK, and from the Center for Pulmonary Vascular Disease at Duke University Medical Center, Durham, NC.

### 2.2. Blood pressure data

This study uses clinically de-identified RHC data from nine patients with confirmed PH: five with PAH and four with CTEPH. Three CTEPH and three PAH datasets are from Duke University, and one CTEPH and two PAH datasets are from the Scottish Pulmonary Vascular Unit. Static data include height (cm), weight (kg), sex (male (m) or female (f)), age (years), heart rate (bpm), and systolic, diastolic and mean systemic blood pressure (mmHg) measured by a blood pressure cuff. The patients underwent RHC, where a catheter is advanced from the right atrium, to the right ventricle, and to the main pulmonary artery. Dynamic pressure waveforms are recorded in each compartment. The pulmonary arterial wedge pressure (PAWP, mmHg), an estimate of left atrial pressure, is also recorded. CO (l min^−1^) is measured during the RHC by thermodilution. All pressure readings are obtained over 7–8 heartbeats. Demographics are provided in table 1.

**Table 1.** Patient demographics; group 1: pulmonary arterial hypertension (PAH); group 4: chronic thromboembolic pulmonary hypertension (CTEPH).

patient	PH	age (years)	sex	height (cm)	weight (kg)	CO (l min^−1^)
1	1	64	male	164.0	72.6	4.0
2	4	58	male	161.0	70.0	4.3
3	1	27	female	151.0	81.1	2.6
4	4	71	female	167.6	93.3	6.1
5	4	51	male	179.1	117.2	3.6
6	1	—	male	178.0	108.0	6.4
7	1	—	male	179.0	74.0	6.3
8	1	—	female	183.0	82.0	5.6
9	4	—	female	154.9	67.4	4.0

CO, cardiac output; PH, pulmonary hypertension. For patients 6–9, age was not included in the medical records.

### 2.3. Data extraction

**Figure 1.**
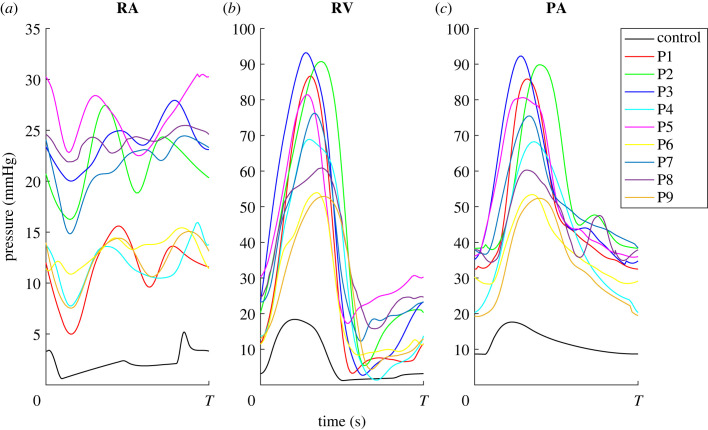
Data processing. Dynamic data from the right atrium (RA), right ventricle (RV) and main pulmonary artery (PA) for each patient are digitized from right heart catheterization recordings and used for model calibration.

Time-series data are extracted from clinical RHC reports using GraphClick v. 3.0.3 for Mac OS and Map Digitizer available on the Apple AppStore. Beat-to-beat haemodynamic profiles for each patient are extracted, and the RHC pressure waveforms are aligned to the electrocardiogram signals. The waveforms are separated by R-R interval and stored as separate files. For this study, a single representative right atrium, right ventricle and main pulmonary artery waveform is chosen for each patient (figure 1). Since RHC data are not measured simultaneously, the representative waveforms are selected during end-expiration and assigned a cardiac cycle length equal to the averaged pressure cycle length. To align the signals within the cardiac cycle, we shift the right atrium and main pulmonary artery signals to ensure that right atrial contraction occurs before the start of right ventricular isovolumic contraction and that peak right ventricular pressure occurs immediately before peak of the pressure in the main pulmonary artery. Magnitudes of the right atrium, right ventricle and main pulmonary artery pressure signals are shifted slightly to ensure physiological valve dynamics. Dynamic pressure waveforms from the RHC are shown in figure 1. Lastly, we construct a normotensive, control patient using pressure and volume values from literature [20,21]; these pressure values are displayed in table 2. Control parameters and model predictions are compared with those obtained using PH data.

**Table 2.** Static pressure values (mmHg) from patient data (denoted by subscript d) used for nominal parameter calculations. Mean and standard deviations are calculated for the PH data only.

data	control	P1	P2	P3	P4	P5	P6	P7	P8	P9	mean ± s.d.
$p_{M,ra}^{d}$	12	14	24	28	16	31	15	24	25	15	21 ± 6
$p_{m,ra}^{d}$	3	5	16	20	8	23	11	15	22	8	14 ± 7
$p_{M,rv}^{d}$	21	87	91	93	69	81	54	76	61	53	74 ± 15
$p_{m,rv}^{d}$	2	3	5	3	1	17	8	12	16	4	8 ± 6
$p_{M,pa}^{d}$	21	86	90	92	68	81	53	75	60	52	73 ± 15
$p_{m,pa}^{d}$	8	32	38	34	20	36	28	37	34	19	31 ± 7
$p_{pa}^{d}$	12	48	55	54	41	53	37	51	45	34	46 ± 8
$p_{W}^{d}$	5^‡^	4	5	8	11	20	10	17	22	12	12 ± 6
$p_{M,sa}^{d}$	120	112	112	127	148	118	133	127	89	123	121 ± 16
$p_{m,sa}^{d}$	80	76	76	90	78	77	87	92	68	65	79 ± 9
$p_{sa}^{d}$	93	88	88	102	101	91	102	103	75	84	93 ± 10

^†^control values obtained from [20,21].

^‡^left atrial diastolic value used in place of PAWP.

### 2.4. Mathematical model

**Figure 2.**
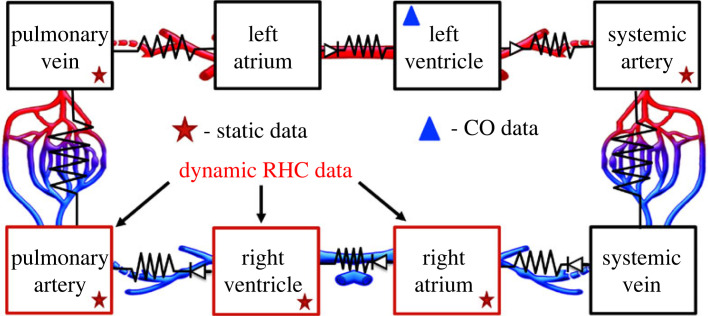
Model schematic. Follows an electrical circuit analogue. The model has eight compartments: the systemic and pulmonary arteries and veins, two atria and two ventricles. Each compartment is modelled as compliant and is separated by a resistor element. The right atrium, right ventricle and pulmonary arteries (red boxes) have both dynamic and static data. The pulmonary veins and systemic arteries have only static data. RHC, right heart catheterization; CO, cardiac output.

This study uses a systems-level, ordinary differential equations (ODE) model (shown in figure 2) that simulates dynamic pressure *p* (mmHg), flow *q* (ml s^−1^) and volume *V* (ml). The model comprises eight compartments: the left and right atria and ventricles, the systemic and pulmonary arteries and veins. The model is formulated using an electrical circuit analogy, with pressure analogous to voltage, flow to current, volume to charge and compliance to capacitance. We include four heart valves, two semilunar (tricuspid and mitral) and two atrioventricular (pulmonary and aortic). An additional systemic venous valve is also included. To ensure proper flow between compartments, heart valves are modelled as diodes, i.e. the valves are either open or closed depending on the pressure gradient between compartments. Equivalent to an RC circuit, equations relating to the three dependent quantities are given by

2.1 $$\frac{dV_{s,i}}{dt}=q_{i-1}-q_{i},$$

2.2 $$\;q_{i}=\frac{p_{i}-p_{i+1}}{R_{i}},$$

2.3 $$\mathrm{and}\;\;\;V_{s,i}=V_{i}-V_{un,i}=C_{i}(p_{i}-p_{i+1}),$$

where the subscripts *i* − 1, *i*, *i* + 1 refer to the prior, current and proceeding compartments in the system. *V*_*s*,*i*_ (ml) denotes the stressed volume (the circulating volume), and *V*_*un*,*i*_ (ml) is the unstressed volume (assumed constant). *R*_*i*_ (mmHg s ml^−1^) denotes the resistance between two compartments, and *C*_*i*_ (ml mmHg^−1^) denotes the compartment compliance. Equation (2.1) ensures conservation of volume, equation (2.2) is the analogue of Ohm’s Law, and equation (2.3) relates volume and pressure.

We model each heart chamber by a time-varying elastance function *E*_*i*_(*t*) (mmHg · ml^−1^) [10,17], which relates pressure and volume by

2.4 $$p_{i}(t)=E_{i}(\tilde{t})V_{s,i},$$

where *i* = *ra*, *la*, *rv*, *lv* denote the left (*l*) and right (*r*) atria (*a*) and ventricles (*v*). The time within the cardiac cycle is denoted by $\tilde{t}=\mathrm{mod}(t,T$) (s), where *T*(s) is the length of the cardiac cycle. The ventricular elastance function $E_{v}(\tilde{t})$ mmHg ml^−1^ is given by the piece-wise continuous function [10]

**Figure 3.**
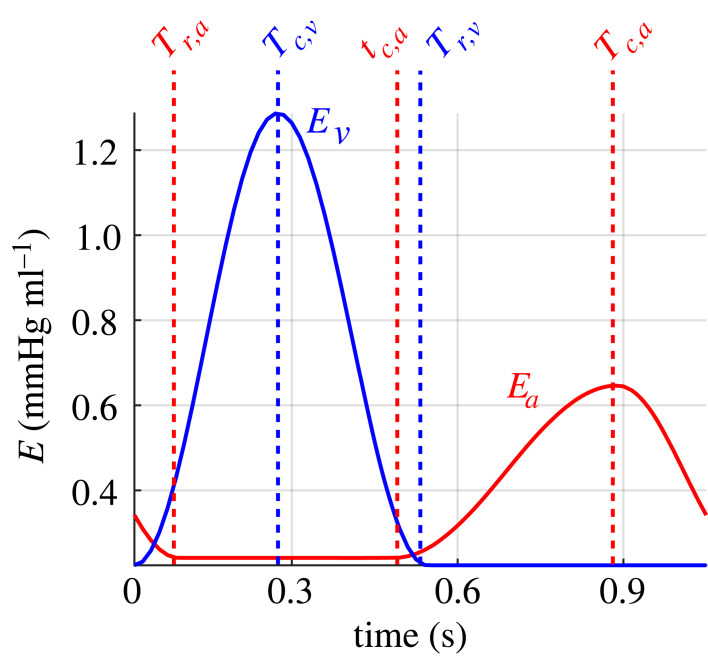
Heart chamber elastance function. Representative elastance function for the atrial (red) and ventricular (blue) heart chambers. Timing parameters are shown above their respective phases of the cardiac cycle. Note that ventricular isovolumic contraction occurs while the atrium is still relaxing.

2.5 $$E_{v}(\tilde{t})=\left\{ \begin{array}{rl} \frac{E_{M,v}-E_{m,v}}{2}\left(\cos\left(\frac{\pi \tilde{t}}{T_{c,v}}\right)\right)+E_{m,v},\; & 0\leq \tilde{t}\leq T_{c,v}\;\\ \frac{E_{M,v}-E_{m,v}}{2}\left(1+\cos\left(\frac{\pi (\tilde{t}-T_{c,v})}{(T_{r,v}-T_{c,v})}\right)\right)+E_{m,v},\; & T_{c,v}<\tilde{t}\leq T_{r,v}\;\\ E_{m,v},\; & T_{r,v}<\tilde{t}\leq T,\; \end{array}\right.$$

where *E*_*m,v*_ and *E*_*M,v*_ (mmHg ml^−1^) are the minimal and maximal ventricular elastances, and *T*_*c*,*v*_ and *T*_*r*,*v*_(s) denote the duration of ventricular contraction and relaxation. The atrial elastance function (shown in figure 3) is prescribed in a similar fashion [22]

2.6 $$E_{a}(\tilde{t})=\left\{ \begin{array}{cc} \frac{E_{M,a}-E_{m,a}}{2}\left(1-\cos\left(\frac{\pi (\tilde{t}-T_{r,a})}{(T-T_{c,a}+T_{r,a}}\right)\right)+E_{m,a},\; & 0\leq \tilde{t}\leq T_{r,a}\;\\ E_{m,a},\; & T_{r,a}<\tilde{t}\leq \tau_{c,a}\;\\ \frac{E_{M,a}-E_{m,a}}{2}\left(1-\cos\left(\frac{\pi (\tilde{t}-\tau_{c,a})}{(T_{c,a}-\tau_{c,a})}\right)\right)+E_{m,a},\; & \tau_{c,a}<\tilde{t}\leq T_{c,a}\;\\ \frac{E_{M,a}-E_{m,a}}{2}\left(1+\cos\left(\frac{\pi (\tilde{t}-T_{c,a})}{(T-T_{c,a}+T_{r,a})}\right)\right)+E_{m,a},\; & T_{c,a}<\tilde{t}\leq T.\; \end{array}\right.$$

Here, *E*_*m,a*_ and *E*_*M,a*_ (mmHg ml^−1^) are the minimum and maximum elastances of the atria and *T*_*r*,*a*_, *τ*_*c*,*a*_ and *T*_*c*,*a*_ (s) denote the start of atrial relaxation, the start of atrial contraction and the point of maximum atrial contraction. The elastance model is parametrized such that $0\leq T_{r,a}\leq \;\tau_{c,a}\leq T_{c,a}\leq T$. Figure 3 shows a representative elastance time course in the atria and ventricles.

### 2.5. Model outcomes

Clinically, PH diagnosis requires invasive measurements of pulmonary blood pressure by RHC. These measurements are typically combined with systolic and diastolic systemic arterial pressure and cardiac output measurements. These measurements describe arterial dynamics but lack detailed information related to ventricular function. Our model predicts pressure, flow and volume in each compartment, augmenting information obtained from measurements. By combining these predictions, we can gain additional insight into the state of the cardiovascular system. Specifically, we compute:

(i) **Stroke work** refers to the integral of the pressure–volume loop
  $$\int_{V}p(t)\;dV^{\prime },$$
  for each heart chamber. This is a clinical measure of ventricular function [9,23,24]. We convert stroke work to Joules (J) using the conversion that 1 J = 7.5 × 10^3^ mmHg ml.
(ii) **Resistance ratio:** the pulmonary and systemic resistance ratio is defined as *R*_*p*_/*R*_*s*_ (non-dimensional) [23].
(iii) **Compliance ratio** of pulmonary and systemic arterial compliance, *C*_*pa*_/*C*_*sa*_ (non-dimensional).
(iv) **Pulsatility index (PI)** refers to to the ratio of pulmonary arterial pulse pressure to average right atrial pressure, $(p_{M,pa}-p_{m,pa})/{\bar{p}}_{ra}$ [25].

### 2.6. Parameter values and initial conditions

The sparse haemodynamic data and many model parameters make it imperative that nominal parameter values and initial conditions are set in a physiologically and patient-specific manner. Following previous approaches [9,17], we use a combination of patient-specific data (where available) and literature values. Table 3 lists the nominal parameter values and their calculation.

**Table 3.** Parameters in the zero-dimensional model and the methods for calculating their nominal values.

parameter	description	units	equation	reference
**heart valves**				
*R*_ava_	aortic valve resistance	mmHg s ml^−1^	(*p*_*M,lv*_ − *p*_*M,sa*_)/*q*_tot_	Ohm’s Law
*R*_mva_	mitral valve resistance	mmHg s ml^−1^	0.01	—
*R*_pva_	pulmonary valve resistance	mmHg s ml^−1^	(*p*_*M,rv*_ − *p*_*M,pa*_)/*q*_tot_	Ohm’s Law
*R*_*tva*_	tricuspid valve resistance	mmHg s ml^−1^	0.03	—
*R*_*sv*_	systemic venous resistance	mmHg s ml^−1^	$({\bar{p}}_{sv}-p_{m,ra})/q_{\mathrm{tot}}$	Ohm’s Law
*R*_*pv*_	pulmonary venous resistance	mmHg s ml^−1^	$({\bar{p}}_{pv}-p_{m,la})/q_{\mathrm{tot}}$	Ohm’s Law
**systemic vasculature**				
*R*_*s*_	systemic resistance	mmHg s ml^−1^	$(p_{m,sa}-{\bar{p}}_{sv})/q_{\mathrm{tot}}$	Ohm’s Law
*C*_*sa*_	systemic arterial compliance	ml mmHg^−1^	(*V*_*M,sa*_ − *V*_*un,sa*_)/*p*_*m,sa*_	[9]
*C*_*sv*_	systemic venous compliance	ml mmHg^−1^	$(V_{M,sv}-V_{un,sv})/{\bar{p}}_{sv}$	[9]
**pulmonary vasculature**				
*R*_*p*_	pulmonary resistance	mmHg s ml^−1^	$(p_{m,pa}-{\bar{\;p}}_{\;pv})/q_{\mathrm{tot}}$	Ohm’s Law
*C*_*pa*_	pulmonary arterial compliance	ml mmHg^−1^	(*V*_*M,pa*_ − *V*_*un,pa*_)/*p*_*m,pa*_	[9]
*C*_*pv*_	pulmonary venous compliance	ml mmHg^−1^	$(V_{\;M,pv}-V_{\;un,pv})/{\bar{\;p}}_{\;pv}$	[9]
**heart elastance**				
*E*_*M*,*rv*_	maximal right ventricular elastance	mmHg ml^−1^	*p*_*M,rv*_/*V*_*m,rv*_ − *V*_*un,rv*_	[17]
*E*_*m*,*rv*_	minimal right ventricular elastance	mmHg ml^−1^	*p*_*m,rv*_/(*V*_*M,rv*_ − *V*_*un,rv*_)	[17]
*E*_*M*,*ra*_	maximal right atrial elastance	mmHg ml^−1^	*p*_*M,ra*_/(*V*_*m,ra*_ − *V*_*un,ra*_)	[17]
*E*_*m*,*ra*_	minimal right atrial elastance	mmHg ml^−1^	*p*_*m,ra*_/(*V*_*M,ra*_ − *V*_*un,ra*_)	[17]
*E*_*M*,*lv*_	maximal left ventricular elastance	mmHg ml^−1^	*p*_*M,lv*_/(*V*_*m,lv*_ − *V*_*un,lv*_)	[17]
*E*_*m*,*lv*_	minimal left ventricular elastance	mmHg ml^−1^	*p*_*m,lv*_/(*V*_*M,lv*_ − *V*_*un,lv*_)	[17]
*E*_*M*,*la*_	maximal left atrial elastance	mmHg ml^−1^	*p*_*M,la*_/(*V*_*m,la*_ − *V*_*un,la*_)	[17]
*E*_*m*,*la*_	minimal left atrial elastance	mmHg ml^−1^	*p*_*m,la*_/(*V*_*M,la*_ − *V*_*un,la*_)	[17]
**heart timing**				
*τ*_*r*,*a*_	beginning of atrial contraction	s	data	—
*T*_*c*,*a*_	peak atrial contraction	s	data	—
*T*_*r*,*a*_	end of atrial systole	s	data	—
*T*_*c*,*v*_	peak ventricular systole	s	data	—
*T*_*r*,*v*_	beginning of ventricular diastole	s	data	—

#### 2.6.1. Compartment volumes and cardiac output

Using Hidalgo’s formula [26], each patients’ total blood volume *V*_tot_ (ml) is calculated as a function of height *H* (cm), weight *W* (kg) and sex [27] as

2.7 $$V_{\mathrm{tot}}=\left\{ \begin{array}{cc} 3.47\cdot \text{BSA}-1.954\cdot 1000,\; & \text{if female}\;\\ 3.29\cdot \text{BSA}-1.229\cdot 1000,\; & \text{if male}\; \end{array}\right.,$$

where $\mathrm{BSA}=\sqrt{W\cdot H/3600}\;(m^{2})$ is the body surface area [28].

The heart’s initial stressed volumes (initial conditions) are calculated using BSA-indexed values. By contrast, stressed volumes in the vasculature are based on blood volume proportions [29]. The BSA-indexed end-diastolic (maximal) volumes $V_{M,i}$ (ml) for the right heart are based on Tello *et al.* [30], with $V_{M,ra}=58.9\cdot \mathrm{BSA}$ and $V_{M,rv}=116.9\cdot \mathrm{BSA}$. We assume that the left heart chamber volumes (also in ml) are unaffected by PH. We assume that $V_{M,la}=30\cdot \mathrm{BSA}$ and $V_{M,lv}=80\cdot \mathrm{BSA}$ [21]. Note that these values determine the blood volume distributions for PH patients. The normotensive control simulation used $V_{M,ra}=30\cdot \mathrm{BSA}$ and $V_{M,rv}=78\cdot \mathrm{BSA}$, $V_{M,la}=30\cdot \mathrm{BSA}$ and $V_{M,lv}=78\cdot \mathrm{BSA}$ [21].

The total volumes for the systemic and pulmonary arteries are 13% and 3% of *V*_tot_, of which the stressed volumes are 27% and 58% of the total volume. Pulmonary venous blood volume is 11% of *V*_tot_, and 11% of this volume is stressed. These values are from previous studies [10,17]. To ensure that blood volume distributions add to 100%, we calculate total systemic venous blood volume as the remaining volume

$$V_{sv\text{\%}}=100-13-3-11-V_{H\text{\%}},$$

where $V_{H\text{\%}}$ is the percentage of total blood volume within the heart. CO is calculated assuming that the total blood volume circulates in 1 min [10,20].

#### 2.6.2. Pressure

Pulmonary circulation pressures are extracted from the RHC data, while the systemic arterial pressure is determined from cuff measurements. These values are listed in table 2. Nominal pressure values for compartments for which we do not have measurements (i.e. the left atrium, left ventricle and systemic veins) are calculated by scaling pressures in their adjacent, data-calibrated compartments [27]. We use the following relationships for compartments for which we do not have data:

2.8 $$p_{sv}=\max(10,1.15\;p_{m,rv}),$$

2.9 $$p_{m,la}=0.95\;p_{\;pv},$$

2.10 $$p_{M,la}=p_{m,la}+5,$$

2.11 $$p_{m,lv}=0.97{\;p}_{M,la},$$

2.12 $$\;p_{M,lv}=1.01\;p_{M,sa}.$$

The subscripts *sa*, *sv*, *la* and *pv* denote the systemic arteries, systemic veins, left atrium and pulmonary veins, respectively. The additional subscripts *m* and *M* denote the minimum and maximum values. We assume a pulse pressure of 5 mmHg for the left atrium, consistent with previous studies [31].

#### 2.6.3. Resistance

Each compartment is separated by a resistance to flow. Using Ohm’s Law, the nominal vascular resistance (mmHg s ml^−1^) is calculated as

2.13 $$R_{i}=\frac{\Delta p}{\text{CO}},$$

where the resistance in compartment *i* depends on the pressure gradient, Δ*p* (mmHg), and the CO; refer to table 3 for more details. The aortic and pulmonary valve resistances are calculated as

2.14 $$R_{\mathrm{ava}}=\frac{\;p_{M,lv}\;-p_{M,sa}}{\text{CO}}\;\text{and}\;R_{\;pva}=\frac{\;p_{M,rv}-p_{\;M,pa}}{\text{CO}}.$$

For PH patients, right atrial and pulmonary venous pressures are elevated [32], and resistance equations overestimate atrioventricular valve resistance. To circumvent this, we set *R*_*tva*_ = 0.03 and *R*_mva_ = 0.01 (mmHg s ml^−1^) for all nine PH patients.

#### 2.6.4. Compliance

The compliance is defined as the relative change in volume for a given change in pressure [33] and quantifies the ability of the vasculature to distend under load. In this study, nominal compliance (ml mmHg^−1^) estimates are

2.15 $$C_{i}=\frac{V_{i}-V_{un,i}}{{\tilde{\;p}}_{i}},$$

where ${\tilde{\;p}}_{i}\;(\mathrm{mmHg})$ is a compartment-specific pressure [9]; see table 3 for more details.

#### 2.6.5. Heart parameters

The heart model includes elastance and timing parameters. Noting that compliance is the inverse of elastance and that the compliance in the heart is minimal during end-systole (computed at the maximum pressure and minimal volume) [17], we calculate the maximum and minimum elastances (mmHg ml^−1^) as

2.16 $$E_{M,i}=\frac{\;p_{M,i}}{V_{m,i}-V_{un,i}}\;\text{and}\;E_{m,\;i}=\frac{\;p_{m,i}}{V_{M,i}-V_{un,i}},$$

where *i* = *la*, *ra*, *lv*, *rv*.

Nominal timing parameters for the right atrial and ventricular elastance functions are extracted from the time-series data. Maximum and minimum right ventricular elastance occur at peak systole and the beginning of diastole, corresponding to *T*_*c*,*v*_ and *T*_*v*,*r*_, respectively. Right atrium data are used to determine the end of atrial systole, the start of atrial contraction and peak atrial contraction, i.e. *T*_*r*,*a*_, *τ*_*c*,*a*_ and *T*_*c*,*a*_. Since dynamic data are unavailable for the left atrium and ventricle, we set left-heart chamber timing parameters equal to the right-heart timing parameters.

### 2.7. Model summary

The model consists of a system of eight ODEs describing the stressed volumes, *V*_*s*,*i*_ (ml), for each compartment, with 25 parameters. The system is written as

2.17 $$\begin{array}{rl} y & =g(t,x;\theta ),\\ \frac{dx}{dt} & =f\;(t,x;\theta ),\\ x & =\{V_{la},V_{lv},V_{sa},V_{sv},V_{ra},V_{rv},V_{\;pa},V_{\;pv}\}, \end{array}\}$$

where

2.18 $$\begin{array}{rl} \theta & =\{R_{s},R_{p},R_{ava},R_{mva},R_{\;pva},R_{tva},R_{\;pv},R_{sv},C_{sa},C_{sv},C_{\;pa},C_{\;pv},\\ & \;E_{M,la},E_{m,la},E_{M,ra},E_{m,ra},E_{M,lv},E_{m,lv},E_{M,rv},E_{m,rv}\\ & \;T_{r,a},\tau_{c,a},T_{c,a},T_{c,v},T_{r,v},\}. \end{array}$$

Here, ***x*** denotes the state variables (*V*_*s*,*i*_ in compartment *i*). The functions *f*(*t*, ***x***; *θ*) denote the evolution of the states (equation (2.1)), and θ are the parameters. The vector ***y*** is the model output computed as function *g*(*t*, ***x***; *θ*) of time, the state variables and the model parameters. The output include predictions of pressure (mmHg) and CO (l min^−1^), used for parameter inference.

### 2.8. Parameter inference

We estimate model parameters, some corresponding to disease biomarkers, by minimizing the least-squares error between model predictions and data. We use the Levenberg–Marquardt algorithm to solve the generalized least-squares problem [34]. The observed data ***y***^*d*^ (static or time series) is assumed to be of the form

2.19 $$y^{d}=g(t,x;\theta )+\varepsilon ,$$

where *g*(*t*, ***x***; *θ*) are the model predictions (here, pressure and CO), and ε is the measurement error, assumed to be independent and identically distributed (iid) white Gaussian noise, i.e. $\varepsilon ∼N(0,\sigma_{\varepsilon }^{2}I)$. Using this framework, we estimate parameters that minimize the sum of squared errors, *J* = ***r***^*T*^***r***, where ***r*** is the weighted residual vector. The residual encompasses the differences between the measured data ***y***^*d*^ and model predictions ***y*** = *g*(*t*, ***x***; *θ*). Since the data have different units and order of magnitude, each residual component is weighted relative to the data.

The static residual (including systolic, diastolic and mean blood pressure in addition to CO values) is defined as

2.20 $$r_{s}=\frac{1}{\sqrt{N_{s}}}\frac{y-y^{d}}{y^{d}},$$

where the vector ***y*** = {*p*_*M,ra*_, *p*_*m,ra*_, *p*_*M,rv*_, *p*_*m,rv*_, *p*_*M,pa*_, *p*_*m,pa*_, *p*_*M,sa*_, *p*_*m,sa*_, *p*_*m,pv*_, CO}, includes model outputs, ***y***^*d*^ is the corresponding data and *N*_*s*_ is the number of points. The three dynamic residuals (used to fit the waveform data) are given by

2.21 $$r_{ra}=\frac{1}{\sqrt{N_{ra}}}\frac{p_{ra}(t;\theta )-p_{ra}^{d}(t)}{p_{ra}^{d}(t)},$$

2.22 $$r_{rv}=\frac{1}{\sqrt{N_{rv}}}\frac{p_{rv}(t;\theta )-p_{rv}^{d}(t)}{p_{rv}^{d}(t)},$$

2.23 $$\;r_{\;pa}=\frac{1}{\sqrt{N_{\;pa}}}\frac{p_{\;pa}(t;\theta )-p_{\;pa}^{d}(t)}{p_{\;pa}^{d}(t)},$$

where $p_{i}(t;\theta ),p_{i}^{d}(t)$ and *N*_*i*_ are the time-series pressure predictions, time-series pressure data and number of residual points for the right atrium, right ventricle, and main pulmonary artery. We consider two combined residuals as our quantity of interest

$$r_{1}=r_{s}$$

and

$$r_{2}=\{r_{s},r_{ra},r_{rv},r_{\;pa}\}.$$

Similar to the approach in [17], each residual is computed over the last 30 cycles of the model predictions. Since we do not have volume data, we include four penalty terms in the inference procedure to constrain heart chamber volumes. PAH and CTEPH patients have an enlarged right heart, increasing the volume of the right atrium and ventricle [30]. We penalize end-diastolic model predictions below a BSA-indexed volume threshold, as defined in §2.6.1. The penalty functions are defined by

2.24 $$J_{\mathrm{penalty},i}=\max\left(0,\frac{V_{M,i}^{d}-\max(V_{i})}{V_{M,i}^{d}}\right),$$

where *i* = *la*, *lv*, *ra*, *rv* and ***V***_*i*_ are the predicted chamber volumes.

### 2.9. Sensitivity analyses

To determine what parameters can be estimated given the model and the available data, we first compute each parameter’s influence on the residual model. To do so, we calculate the sensitivity of the residual vectors ***r***_**1**_ and ***r***_**2**_ with respect to the model parameters.

Both local, derivative-based, and global, variance-based, sensitivity analyses are used. The former methods are valid within a small neighbourhood of the nominal parameter values and quantify the gradient of the residual vectors ***r***_**1**_ and ***r***_**2**_ with respect to the parameters. The latter measures the sensitivity throughout the physiological parameter space, simultaneously varying multiple factors. Both methods provide valuable and distinct information. Therefore, we combine local and global sensitivity analysis to identify non-influential parameters. These are fixed at their nominal values before subset selection.

The local sensitivity of the residual for a parameter *θ*_*i*_ at time *t* is denoted by *χ*_*i*_(*t*). Sensitivities are approximated numerically via the complex-step method [35]. We rank parameters from most to least influential by calculating the 2-norm of each sensitivity [9,17]

2.25 $$\|\chi_{i}(t){\|}_{2}^{2}={\left(\sum_{l=1}^{N}\chi_{i}^{2}(t_{l})\right)}^{1/2},$$

where i=1,2,…,M is the number of parameters and *l* = 1, 2, …, *N* is the length of the time vector.

While global sensitivity analysis is more computationally expensive than local methods, its ability to vary multiple parameters at a time may expose undiscovered relationships between parameters [12]. In this study, we use variance-based global sensitivity analysis methods, computing first (*S*_*i*_) and total order ($S_{T_{i}}$) Sobol’ indices [36]). The former measures the parameters’ individual contribution to the total output variance of the cost function, and the latter the individual contributions and higher-order interactions between the parameters on the variance. $S_{T_{i}}$ are used to order parameters from most to least influential. A more detailed description of the local and global methods is given in Section S2 of the electronic supplementary material.

### 2.10. Parameter subset selection

Once the sensitivity analysis is performed, additional steps are taken to determine if the parameters are practically identifiable, i.e. unique parameter estimates are associated with the minimum of our objective function [37,38]. The model used in this study is analogous to an electrical resistor–capacitor circuit. Circuit theory dictates that resistors and capacitors in series and parallel can be combined to give an equivalent resistor and capacitor. Therefore, if no data is available between two components, their parameters cannot be estimated uniquely, i.e. they are non-identifiable. Given the limited data and a large number of parameters (found in (2.18)), we expect identifiability problems if all parameters are inferred from data [9,17]. We take several steps to determine an identifiable and influential subset with respect to the residual vectors. The subset selection process begins by analysing the global sensitivity results. Parameters with $S_{T_{i}}\approx 0$ are considered non-influential and fixed at their nominal values [12,39]. After excluding these parameters, we use a singular value decomposition (SVD) QR factorization method to determine local pairwise parameter interactions [37]. Lastly, we use multi-start inference to reduce the subset further until we mitigate all identifiability issues.

#### 2.10.1. Singular value decomposition-QR

The SVD-QR method [40] decomposes the sensitivity matrix as $\chi =U\Sigma V^{⊤}$, where U is the matrix of left orthonormal eigenvectors, Σ is a diagonal matrix of singular values and V is the matrix of right orthonormal eigenvectors. The total number of identifiable parameters, *ρ*, is the numerical rank of Σ and is used to partition V as $V=[V_{\rho }\;V_{P-\rho }]$. The permutation matrix $\tilde{P}$ is determined by QR factorization such that $V_{\rho }^{⊤}\tilde{P}=QR$. Here, Q is an orthogonal matrix, and the first *ρ* columns of R form an upper triangular matrix consisting of diagonal entries in decreasing order. The first *ρ* entries of $\tilde{P}$ establish the identifiable parameters for the subset.

#### 2.10.2. Multi-start inference

The previous methods ensure that the parameters are locally and linearly identifiable. However, they do not guarantee practically identifiable parameter subsets if the model has nonlinear behaviour in output space [39]. Thus, we determine our final subset by inferring parameters from multiple initial guesses randomly selected between ± 20% of the nominal values. Non-identifiable parameters probably approach different values, whereas identifiable parameters, converge to the same value regardless of initial guess [16]. We assess identifiability by calculating each patient’s coefficient of variance (CoV; the standard deviation relative to the mean) for each parameter after multi-start inference. Subsets that exhibit parameter CoV> 10% are reduced by fixing the least influential parameter above this threshold. The multi-start inference is iteratively run until the CoV for each parameter is below the 10% threshold.

### 2.11. Confidence and prediction intervals

Model parameter and output uncertainty are quantified using asymptotic analyses [41]. Under the assumption that the noise ε is iid, we compute the variance estimator ${\hat{\sigma }}_{\epsilon }^{2}$ and parameter covariance estimator $\hat{C}={\hat{\sigma }}_{\epsilon }^{2}{\left({\hat{\chi }}^{⊤}\hat{\chi }\right)}^{-1}$ using asymptotic analysis for nonlinear least-squares [42]. We note that asymptotic analyses use the model outputs instead of the residual vector; hence χ here is the sensitivity of the model outputs.

The 95% parameter confidence intervals for each inferred parameter, ${\hat{\theta }}_{i}$, are computed as

2.26 $$[{\hat{\theta }}_{i}^{CI-},{\hat{\theta }}_{i}^{CI+}]={\hat{\theta }}_{i}\pm t_{N-\rho }^{0.975}\sqrt{{\hat{C}}_{i,i}},$$

where $t_{N-M^{\prime }}^{1-\alpha /2}$ is a two-sided *t*-statistic with a 1−α/2= 95% confidence level, and $\sqrt{{\hat{C}}_{i,i}}$ represents the standard error for the *i*th parameter estimator. Throughout we denote these confidence intervals by mean ± 2 s.d., i.e. ${\hat{\theta }}_{i}\pm 2\sigma_{\theta_{i}}$. The confidence and prediction intervals for the optimal model output ${\hat{y}}_{j}$ at time *t*_*j*_ are given by

2.27 $$[{\hat{y}}_{j}^{CI-},{\hat{y}}_{j}^{CI+}]={\hat{y}}_{j}\pm t_{N-\rho }^{0.975}\sqrt{{\hat{\chi }}_{\;j}^{T}{\hat{C}}_{i,i}{\hat{\chi }}_{\;j}}$$

and

2.28 $$[{\hat{y}}_{j}^{PI-},{\hat{y}}_{j}^{PI+}]={\hat{y}}_{j}\pm t_{N-\rho }^{0.975}\sqrt{\sigma_{\varepsilon }^{2}+\;{\hat{\chi }}_{\;j}^{T}{\hat{C}}_{i,i}\;{\hat{\chi }}_{\;j}},$$

where ${\hat{\chi }}_{j}^{T}$ is the sensitivity vector at *t*_*j*_ evaluated at $\hat{\theta }=\{{\hat{\theta }}_{\rho },\theta_{M-\rho }\}$. Note that the prediction intervals account for the variance in both the model output and the data, hence they are wider.

### 2.12. Simulations

To study the impact of PH, we run several simulations comparing PH patients with a normotensive control subject.

*Control:* Simulations for a control patient are conducted using normotensive pressure and volume values given in table 2. Haemodynamic predictions are compared with those from PH patients.

*Static:* Similar to Colunga *et al.* [9], we calibrate model predictions using only static pressure and CO data for each PH patient, i.e. ***r***_1_. We use this as a benchmark procedure to determine the effects of adding dynamic waveforms.

*Dynamic waveforms:* Model predictions of systolic, diastolic and mean pressure are computed in combination with dynamic right atrium and ventricle and pulmonary artery predictions using residual ***r***_2_.

## 3. Results

Local and global sensitivity analyses of both residuals ***r***_1_ and ***r***_2_ distinguish influential and non-influential parameters. Next, SVD-QR and multi-start inference are used to construct subsets of identifiable parameters. Model predictions are calibrated to measured RHC data using the identifiable subset, and other outcomes, such as pressure–volume loops, are computed. Uncertainty of parameter estimates and model outputs are compared for the two residual vectors and are shown here for a single representative patient; results for the remaining patients are given in the electronic supplementary material.

### 3.1. Sensitivity analyses

**Figure 4.**
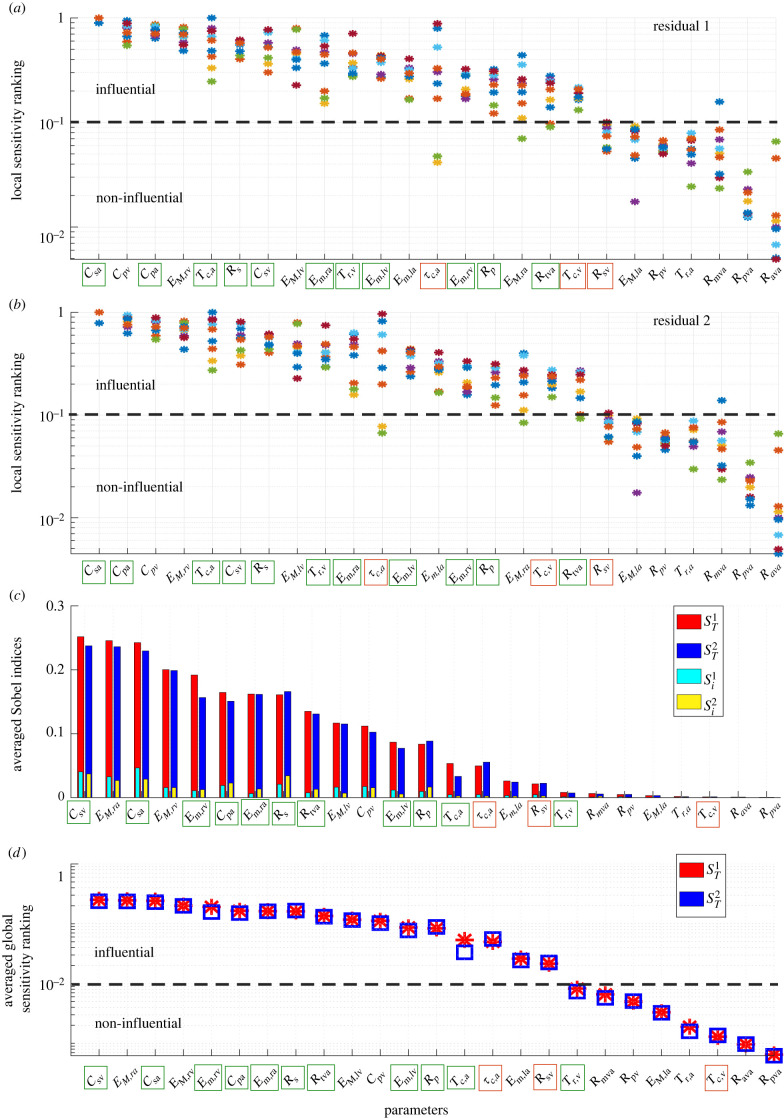
Sensitivity analysis. Parameter ranking based on a local sensitivity analysis using either $r_{1}$ (*a*) or $r_{2}$ (*b*). Each patient is plotted by a different colour. The parameter order is based on median sensitivity across all patients. Average first order (*S*_*i*_) and total order $\left(S_{T_{i}}\right)$ Sobol’ indices using either $r_{1}$ or $r_{2}$ (*c*). Average parameter ranking based on $\left(S_{T_{i}}\right)$ magnitude for either residual are shown in (*d*). The horizontal dashed lines separate influential (above) and non-influential (below) parameters. Green boxes highlight parameters used for ***r***_1_ and red boxes are additional parameters included in ***r***_2_.

Figure 4*a*,*b* shows the patient-specific local sensitivity parameter ranking for ***r***_1_ (static values only, *a*) and ***r***_2_ (static and time-series data, *b*). Sensitivities are normalized by the largest magnitude for each patient and residual, and parameters are sorted based on their median ranking across all nine patients.

Parameters are ranked similarly for the two residual vectors; however, accounting for dynamic predictions makes the timing parameter *τ*_*c*,*a*_ more influential on ***r***_2_. The most influential parameters for both residuals are *C*_*sa*_, *C*_*pa*_, *C*_*pv*_ and *E*_*M*,*rv*_. Seven of the nine patients display consistent parameter rankings for both residual vectors. Parameter *τ*_*c*,*a*_ is less influential for patients 3 and 5 than for the other patients. Overall, the local analysis shows that resistances *R*_*ava*_, *R*_*mva*_, *R*_*pva*_, *R*_*pv*_, the elastance *E*_*M*,*la*_ and the timing parameter *T*_*r*,*a*_ are below the dashed line denoting a normalized sensitivity ≤10^−1^ in figure 4. We further examine these parameters using global sensitivity analysis to determine if they are non-influential.

For the global sensitivity analysis, *n* = 10^4^ samples are generated for each parameter using a Sobol’ sequence. This sample size provided robust results in parameter ranking when compared with 5 × 10^4^ samples (results not shown). The average first order (*S*_*i*_) and total ($S_{T_{i}}$) effects across all nine patients are shown in figure 4*c*,*d* for the cost functional *J*(*θ*) using residuals ***r***_1_ and ***r***_2_. Sobol’ indices are similar across all patients, and the parameter ranking using the total Sobol’ index agrees with the local results. A total index, $S_{T_{i}}$, near zero (≤10^−2^) suggests that the corresponding parameter is non-influential. Results show that $S_{T_{i}}$ is ≤0.005 for parameters *R*_*ava*_, *R*_*pva*_, *R*_*pv*_, *R*_*mva*_, *E*_*M*,*la*_ and *T*_*r*,*a*_, consistent with the local sensitivity results, suggesting that these parameters can be fixed at their nominal values. The $S_{T_{i}}$ is also approximately zero for *T*_*c*,*v*_ and *T*_*r*,*v*_. Since the local sensitivity marked *T*_*c*,*v*_ and *T*_*r*,*v*_ as influential, we include these in our subset selection procedure.

### 3.2. Parameter subsets and inference

Both SVD-QR and multi-start inference are used for parameter subset selection. The non-influential parameters, *θ*^*NI*^ = {*R*_*ava*_, *R*_*mva*_, *R*_*pva*_, *R*_*pv*_, *E*_*M*,*la*_, *T*_*r*,*a*_} are fixed prior to SVD-QR. Previous studies [27] found that the maximum and minimum elastance cannot be inferred simultaneously. Since the minimum elastance affects both the amplitude and baseline elastance, this parameter contains more information and is, therefore, more important to infer. The study by Domogo & Ottesen [43] focused on left atrial dynamics using a zero-dimensional computational model. They found that changes in atrial volume are sensitive to maximal atrial compliance (i.e. minimal atrial elastance). This observation supports our exclusion of maximal elastance parameters in subset selection. The remaining maximal elastances, {*E*_*M*,*ra*_, *E*_*M*,*rv*_, *E*_*M*,*lv*_}, are also fixed prior to SVD-QR. We generate a subset for each residual, including parameters consistently identified by SVD-QR across all nine patients. Parameters that are inconsistent using SVD-QR are depicted in blue in tables S1 and S2 of the electronic supplementary material.

We run the multi-start inference with these reduced SVD-QR subsets. For instances of multi-start inference that have parameters with a high CoV( >0.10) (purple in tables S1 and S2 in the electronic supplementary material), the least influential parameter is removed from the subset and fixed at its nominal value. The final subsets used for each residual are

3.1 $$\theta^{\;r_{1}}=\{R_{s},R_{p},R_{tva},C_{sa},C_{sv},C_{\;pa},E_{m,ra},E_{m,rv},E_{m,lv},T_{c,a},T_{r,v}\}$$

and

3.2 $$\begin{array}{rl} \theta^{\;r_{2}} & =\{R_{s},R_{p},R_{tva},R_{sv},C_{sa},C_{sv},C_{\;pa},E_{m,ra},E_{m,rv},\\ & \;E_{m,lv},\tau_{c,a},T_{c,a},T_{c,v},T_{r,v}\}. \end{array}$$

**Figure 5.**
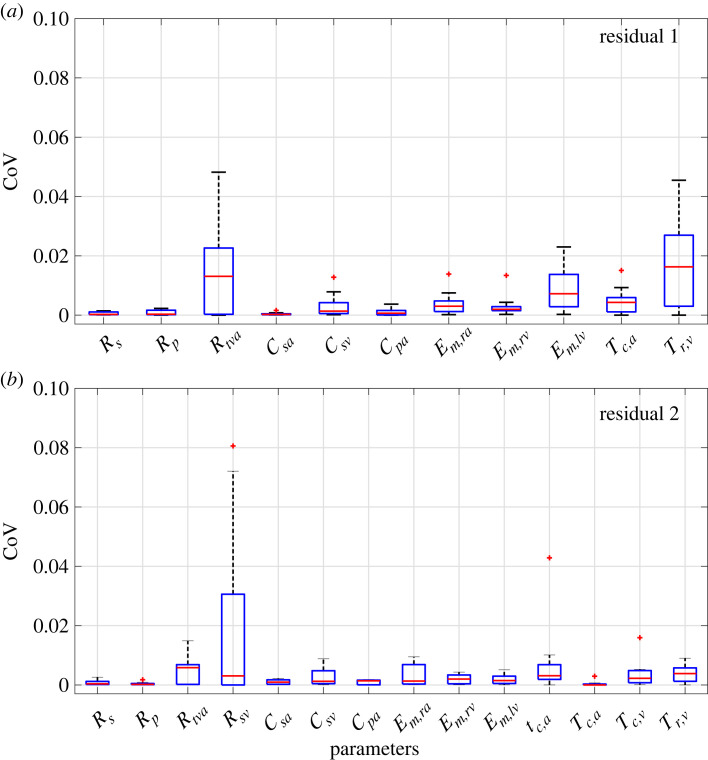
Multi-start Inference. For each patient, each subset is tested for identifiability using eight randomized starting guesses within ±20% of the nominal value. Coefficient of variance (CoV) for the final parameter sets using (*a*) $r_{1}$ or (*b*) $r_{2}$ provide a CoV below 10%.

Figure 5 shows the CoV of the final subsets for ***r***_1_ and ***r***_2_. Tables 4 and 5 list the estimated parameters using either ***r***_1_ or ***r***_2_. These optimal values reflect the optimization starting from the nominal guesses for each patient. We also calculate the 95% parameter confidence intervals using equation (2.26).

**Table 4.** Estimated parameter values using ***r***_1_ along with the 95% confidence interval (depicted as ${\hat{\theta }}_{i}\pm 2\sigma_{\theta_{i}}$). Note that the units for parameters are resistances *R_i_* (mmHg s ml^−1^), compliance *C_i_* (ml mmHg^−1^), elastance *E_i_* (mmHg ml^−1^) and time scales *T_i_* (s).

*θ*	P1	P2	P3	P4	P5	P6	P7	P8	P9
*R*_*s*_	0.82 ± 0.14	1.13 ± 0.97	1.2 ± 0.65	1.14 ± 0.12	1.08 ± 0.82	0.9 ± 0.12	0.86 ± 0.16	0.55 ± 0.74	1.24 ± 0.12
*R*_*p*_	0.5 ± 0.11	0.92 ± 0.71	0.77 ± 0.42	0.33 ± 0.21	0.59 ± 0.87	0.28 ± 0.18	0.36 ± 0.22	0.26 ± 0.71	0.33 ± 0.18
*R*_*tva*_	0.02 ± 1.63	0.12 ± 3.95	0.12 ± 2.26	0.07 ± 0.57	0.09 ± 3.01	0.01 ± 3.29	0.01 ± 2.03	0.03 ± 1.57	0.05 ± 0.65
*C*_*sa*_	2.11 ± 0.02	0.99 ± 0.08	1.45 ± 0.08	0.86 ± 0.02	0.98 ± 0.09	1.69 ± 0.02	2.18 ± 0.02	2.37 ± 0.06	0.67 ± 0.02
*C*_*sv*_	57.86 ± 0.36	22.36 ± 0.7	14.26 ± 0.48	45.68 ± 0.45	21.16 ± 0.87	34.67 ± 0.18	15.67 ± 0.13	13.4 ± 0.41	35.67 ± 0.25
*C*_*pa*_	1.33 ± 0.01	0.63 ± 0.08	0.82 ± 0.06	1.13 ± 0.03	0.82 ± 0.1	2.95 ± 0.02	1.84 ± 0.02	1.69 ± 0.06	0.98 ± 0.03
*E*_*m,ra*_	0.06 ± 0.37	0.23 ± 0.71	0.28 ± 0.48	0.1 ± 0.44	0.25 ± 0.81	0.1 ± 0.18	0.16 ± 0.13	0.26 ± 0.39	0.12 ± 0.25
*E*_*m,rv*_	0.03 ± 0.62	0.03 ± 2.18	0.02 ± 3.51	0.01 ± 3.36	0.07 ± 0.83	0.05 ± 0.64	0.09 ± 0.1	0.09 ± 0.51	0.03 ± 0.56
*E*_*m,lv*_	0.02 ± 0.13	0.03 ± 2.66	0.04 ± 1.2	0.07 ± 0.15	0.11 ± 0.51	0.05 ± 0.35	0.1 ± 0.29	0.13 ± 0.76	0.09 ± 0.14
*T*_*c*,*a*_	0.73 ± 0.31	0.74 ± 0.54	0.9 ± 0.68	0.9 ± 0.23	0.85 ± 0.91	0.83 ± 0.85	0.86 ± 0.44	0.67 ± 0.94	0.79 ± 0.13
*T*_*r*,*v*_	0.48 ± 0.04	0.52 ± 2.22	0.76 ± 0.85	0.5 ± 0.48	0.6 ± 1.19	0.56 ± 0.63	0.58 ± 0.07	0.54 ± 0.62	0.56 ± 0.32

**Table 5.** Estimated parameter values using ***r***_2_ along with the 95% confidence interval (depicted as ${\hat{\theta }}_{i}\pm 2\sigma_{\theta_{i}}$). In this table, units are resistances *R*_*i*_ (mmHg s ml^−1^), capacitors *C*_*i*_ (ml mmHg^−1^), elastances *E*_*i*_ (mmHg ml^−1^) and timing parameters *T*_*i*_ (s).

Θ	P1	P2	P3	P4	P5	P6	P7	P8	P9
*R*_*s*_	0.77 ± 0.82	1.14 ± 1.31	1.25 ± 0.95	1.12 ± 0.32	1.15 ± 0.57	0.9 ± 0.2	0.79 ± 0.42	0.58 ± 0.28	1.21 ± 0.21
*R*_*p*_	0.49 ± 0.1	0.9 ± 0.14	0.76 ± 0.13	0.34 ± 0.1	0.58 ± 0.12	0.28 ± 0.07	0.36 ± 0.09	0.26 ± 0.05	0.33 ± 0.09
*R*_*tva*_	0.03 ± 0.36	0.12 ± 0.45	0.12 ± 0.3	0.05 ± 0.21	0.09 ± 0.31	0.01 ± 0.25	0.03 ± 0.23	0.03 ± 0.1	0.05 ± 0.19
*R*_*sv*_	0.02 ± 1.77	0.02 ± 2.77	0.01 ± 3.42	0.01 ± 1.84	0.01 ± 1.69	0.01 ± 1.07	0.05 ± 1.03	0.01 ± 0.58	0.02 ± 1.11
*C*_*sa*_	2.04 ± 0.12	0.99 ± 0.11	1.5 ± 0.14	0.85 ± 0.05	1.01 ± 0.06	1.68 ± 0.03	2.12 ± 0.06	2.43 ± 0.04	0.66 ± 0.03
*C*_*sv*_	35.45 ± 0.47	23.24 ± 0.23	15.7 ± 0.13	42.86 ± 0.21	24.05 ± 0.08	33.5 ± 0.1	12.16 ± 0.2	14.42 ± 0.02	31.12 ± 0.16
*C*_*pa*_	1.3 ± 0.02	0.63 ± 0.02	0.89 ± 0.02	1.13 ± 0.02	0.83 ± 0.02	2.91 ± 0.01	1.79 ± 0.01	1.73 ± 0.01	0.95 ± 0.01
*E*_*m,ra*_	0.07 ± 0.25	0.22 ± 0.25	0.24 ± 0.2	0.11 ± 0.16	0.22 ± 0.12	0.1 ± 0.07	0.19 ± 0.09	0.23 ± 0.06	0.13 ± 0.12
*E*_*m,rv*_	0.03 ± 0.38	0.03 ± 0.45	0.02 ± 0.97	0.01 ± 0.8	0.08 ± 0.08	0.05 ± 0.05	0.1 ± 0.06	0.09 ± 0.02	0.03 ± 0.16
*E*_*m,lv*_	0.02 ± 0.44	0.03 ± 0.61	0.04 ± 0.53	0.06 ± 0.22	0.11 ± 0.19	0.05 ± 0.08	0.11 ± 0.17	0.14 ± 0.09	0.09 ± 0.17
*τ*_*c*,*a*_	0.48 ± 0.28	0.43 ± 0.43	0.63 ± 0.22	0.69 ± 0.1	0.41 ± 0.3	0.6 ± 0.09	0.54 ± 0.16	0.44 ± 0.08	0.55 ± 0.11
*T*_*c*,*a*_	0.91 ± 0.14	0.74 ± 0.17	0.92 ± 0.12	0.9 ± 0.08	0.85 ± 0.1	0.87 ± 0.05	0.98 ± 0.05	0.69 ± 0.03	0.82 ± 0.06
*T*_*c*,*v*_	0.31 ± 0.04	0.27 ± 0.05	0.2 ± 0.06	0.29 ± 0.03	0.22 ± 0.04	0.34 ± 0.02	0.35 ± 0.03	0.24 ± 0.02	0.32 ± 0.02
*T*_*r*,*v*_	0.5 ± 0.03	0.51 ± 0.04	0.76 ± 0.04	0.62 ± 0.03	0.48 ± 0.04	0.56 ± 0.02	0.58 ± 0.02	0.51 ± 0.01	0.59 ± 0.02

**Figure 6.**
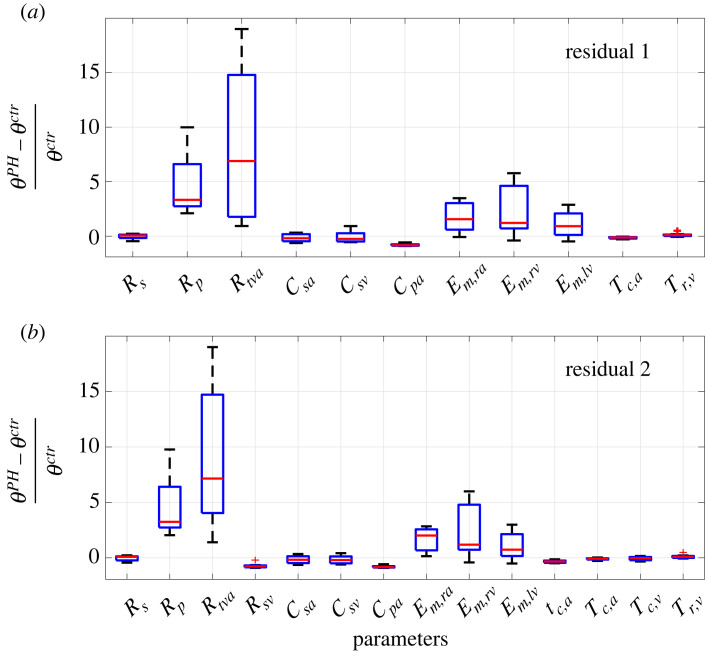
Changes in parameters due to PH. Box and whisker plots showing quantiles and outliers for the estimated parameters. Results show the relative difference from the normotensive parameters.

**Figure 7.**
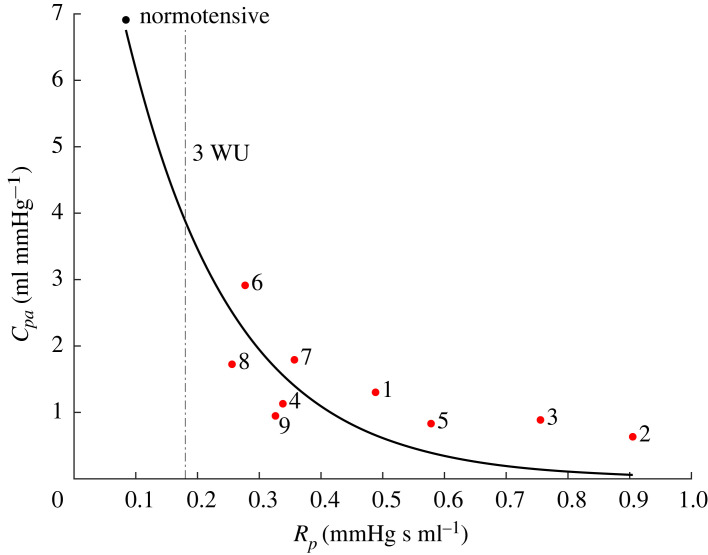
Hyperbolic *R*_*p*_-*C*_*pa*_ relationship. Optimal values of *R*_*p*_ and *C*_*pa*_ for the normotensive (black) and PH (red) patients. The best-fit curve is given by *C*_*pa*_ = 0.6518/(0.1005 + *R*_*p*_), and is similar to previous findings using isolated Windkessel models [44]. A PVR ≥ 3 Wood units (3 WU=0.18, mmHg s ml^−1^) is considered in the PH range (dashed line).

We display the relative change between estimated PH and normotensive parameters in figure 6 as box-and-whisker plots to understand how parameters change with PH. Note that estimated parameters shared between $\theta^{\;r_{1}}$ and $\theta^{\;r_{2}}$ are nearly identical even with additional parameters in $\theta^{\;r_{2}}$. Parameters *R*_*p*_, *R*_*tva*_, *E*_*m*,*ra*_, *E*_*m*,*rv*_ and *E*_*m*,*lv*_ are consistently elevated in all PH patients. The normotensive value of *R*_*tva*_ is substantially smaller than the PH patients, which explains the larger relative change compared with other parameters in the subset. The timing parameters for the heart chambers, compartment compliance and systemic resistance *R*_*s*_ and *R*_*sv*_ remain relatively close to normotensive values. The *R*_*p*_ · *C*_*pa*_ (RC) relaxation times were determined from the inferred parameters. As shown in figure 7, there is a clear inverse relationship between *R*_*p*_ and *C*_*pa*_ with the curve of best fit being *C*_*pa*_ = 0.6518/(0.1005 + *R*_*p*_), *R*^2^ = 0.77, and constant RC relaxation time *R*_*p*_ · *C*_*pa*_ = 0.55 ± 0.15 (s).

### 3.3. Model forecasts and uncertainty

**Figure 8.**
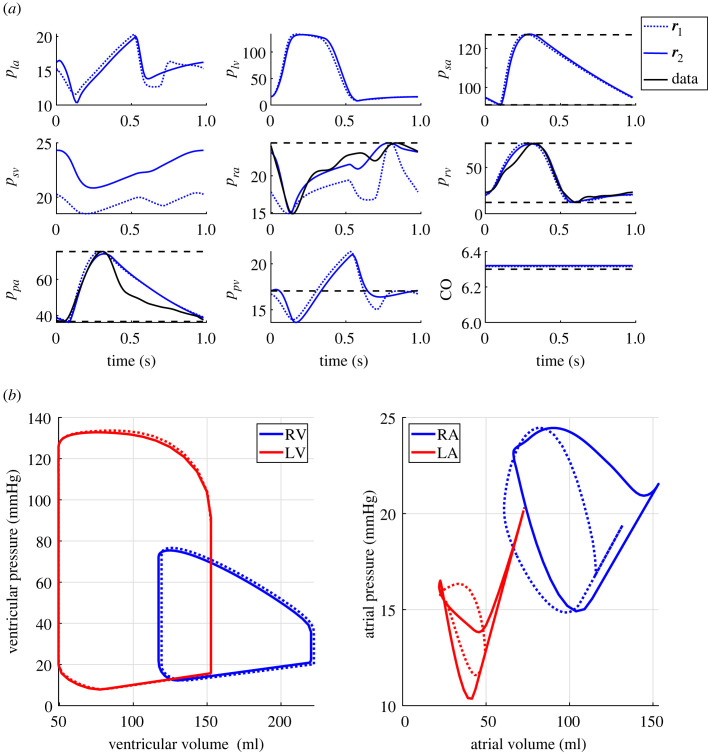
Optimal model predictions. (*a*) Optimal model fits for pressure, *p*_*i*_ (mmHg) and cardiac output, CO (l min^−1^) using either $r_{1}$ (dotted line) or $r_{2}$ (solid line) compared with the data for patient 7. (*b*) simulated pressure-volume loops in the ventricles and atria using residual 1 (dotted line) and residual 2 (solid line) for the dataset from patient 7.

Post-inference predictions of pressure and CO using either $r_{1}$ or $r_{2}$ are depicted in figure 8*a* along with the measured data from patient 7. Predictions for all PH patients are included in the electronic supplementary material. Both $r_{1}$ and $r_{2}$ inference procedures can match the static data well. Using $r_{2}$ minimizes the mismatch between the dynamic model outputs and the time-series data. Predictions of the right atrial dynamics improve drastically when including time-series data. By contrast, right ventricular and main pulmonary artery predictions improve only marginally. For five patients, CO predictions are only slightly worse when matched using $r_{2}$ versus $r_{1}$. However, the maximum and minimum pressure values still match the data well.

**Figure 9.**
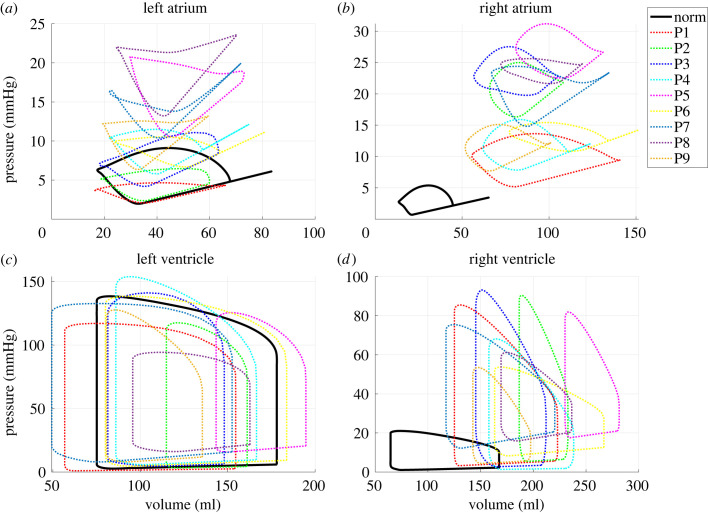
Simulated pressure–volume loops. Pressure–volume loops in the normotensive (norm) and all nine PH patients are contrasted. Model predictions include (*a*) left atrial, (*b*), right atrial, (*c*) left ventricular and (*d*) right ventricular pressure–volume loops.

A benefit of computational models is that essential but unmeasurable outcomes, such as pressure–volume loops, can be predicted. We contrast pressure–volume loops from all four heart chambers for the normotensive subject to the nine PH patients (using estimated parameters from ***r***_2_) in figure 9. Except for patients 1 and 2, all PH patients have increased left atrial pressure. By contrast, the right atrial pressure–volume loops display higher volumes and pressures than the normotensive simulation. The right and left ventricular pressure–volume loops have similar shapes. Yet, the right ventricular pressure–volume loops in the PH group have a more drastic rise in pressure during isovolumic contraction compared with the normotensive results.

We calculate stroke work for all four heart chambers by integrating simulated ventricular pressure predictions with respect to volume. These results and other model outcomes, including the resistance and compliance ratios, *R*_*p*_/*R*_*s*_ and *C*_*pa*_/*C*_*sa*_, and the pulsatility index PI, are shown in table 6. Left atrial stroke work is lower in PH for all but patient 5, and right atrial stroke work is higher in all PH patients relative to the normotensive value. All PH patients have a lower left ventricular stroke work and an elevated right ventricular stroke work relative to the normotensive value. In general, there is a drastic increase in *R*_*p*_/*R*_*s*_ and a decrease in *C*_*pa*_/*C*_*sa*_ in PH relative to normotensive conditions. The pulsatility (PI) decreased in PH except in patient 1.

**Table 6.** Model outcomes from normotensive and PH simulations.

	stroke work (J)						
patient	LA	LV	RA	RV	*R_p_/R_s_*	$C_{\;pa}/C_{sa}$	PI
norm	0.031	1.676	0.013	0.223	0.08	3.84	4.25
1	0.009	1.416	0.058	0.802	0.63	0.64	5.37
2	0.014	0.642	0.031	0.393	0.79	0.64	2.40
3	0.023	1.127	0.033	0.605	0.60	0.59	2.52
4	0.017	1.352	0.035	0.531	0.30	1.33	3.94
5	0.032	0.662	0.037	0.313	0.50	0.83	1.63
6	0.012	1.676	0.034	0.474	0.31	1.74	1.87
7	0.009	1.586	0.042	0.627	0.45	0.84	1.76
8	0.027	0.640	0.016	0.303	0.44	0.71	1.07
9	0.017	0.728	0.028	0.267	0.27	1.44	2.85

Indices include stroke work (joules) in all four heart chambers, resistance ratios (dimensionless), compliance ratios (dimensionless) and pulsatility index (PI, dimensionless) calculated after estimating parameters using $r_{2}$. LA, left atrium; LV, left ventricle; RA, right atrium; RV, right ventricle.

**Figure 10.**
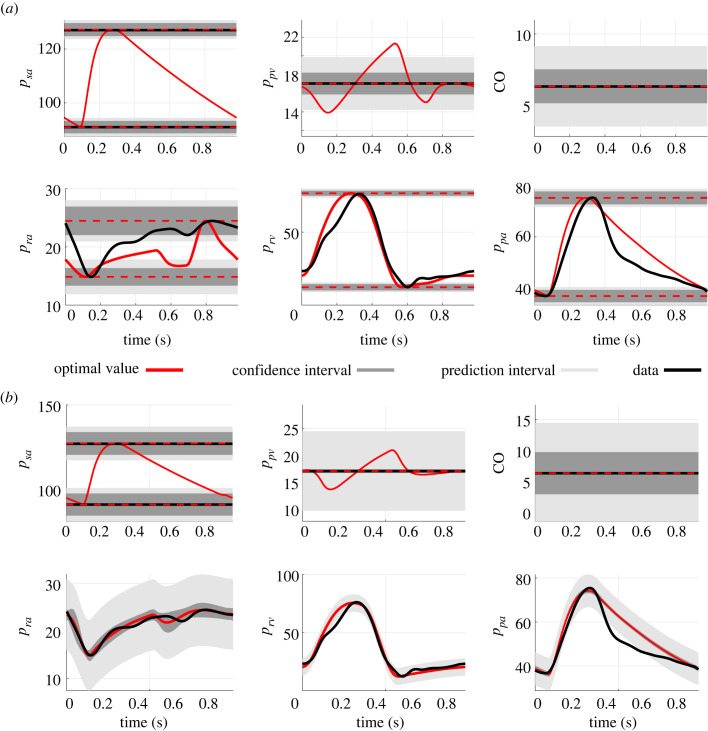
Output uncertainty. Uncertainty in the model outputs for pressure, *p*_*i*_ (mmHg) and cardiac output, CO (l min^−1^) using either $r_{1}$ (*a*) or $r_{2}$ (*b*) for the quantity of interest.

Parameters confidence intervals are provided in table 5. Model confidence and prediction intervals for patient 7 are shown in figure 10 (see the electronic supplementary material for results from all nine patients) using either residual vector. The confidence and prediction intervals show uncertainty in mean pulmonary venous pressure (matched to PAWP data), CO, and systolic and diastolic pressures in the systemic arteries, right atrium, right ventricle and the main pulmonary artery. The confidence intervals for the right ventricle and main pulmonary artery are smaller than the ones for the right atrium. We attribute this discrepancy to the more significant mismatch between right atrium data and model predictions. Adding dynamic data in ***r*_2_** increases the magnitude of the sum of squared residuals, thus increasing the prediction intervals in figure 10*b*. Note that the right atrium, right ventricle and main pulmonary artery data nearly all fall within the 95% prediction intervals shown in figure 10*b*.

## 4. Discussion

Electronic health records typically include RHC blood pressure measurements, estimates of cardiac output, and systemic systolic and diastolic blood pressure measurements. Traditionally, static pressures (e.g. systolic and diastolic) are recorded, though the RHC also generates blood pressure waveforms. Our goal is to examine if additional waveform data improve model calibration and, therefore, characterization of PH and its phenotypes. We use a systems-level cardiovascular model to characterize patient-specific changes due to PH. We use a combination of sensitivity analyses, subset selection and multi-start inference to determine informative and identifiable parameter subsets and estimate these parameters to patient RHC data. Results show that the proposed model captures the hallmarks of PH both with and without waveform data. We find increased right atrial, right ventricular and main pulmonary artery pressures, elevated pulmonary vascular resistance and reduced pulmonary arterial compliance in all PH patients. In addition, we show that additional waveform data are essential in quantifying the right atrial reservoir and pump function. Overall, our results show that systems-level models can capture patient-specific PH dynamics and parallel the current clinical understanding of the disease.

### 4.1. Sensitivity analyses

Sensitivity analysis is crucial for determining which parameters influence the model output. Our model has 25 parameters, yet limited data and the structure of the model make inferring all the parameters infeasible. We use local and global sensitivity analyses on two residual vectors: one comparing static outputs and another static and dynamic outputs. Both methods consistently identify six non-influential parameters independent of the technique and residual. The systemic venous resistance *R*_*sv*_ and timing parameters *T*_*c*,*v*_ and *T*_*r*,*v*_ are not consistently influential across the two techniques. The influential parameters are candidates to be inferred, while the non-influential parameters are fixed at their nominal value.

The pulmonary valve resistance *R*_*pva*_ is non-influential; this parameter is directly associated with the coupling between the right ventricle and the main pulmonary artery. However, none of the PH patients in this study have a history of pulmonary valve stenosis. Thus it is reasonable to keep this parameter fixed at its nominal value. The pulmonary venous *R*_*pv*_ and mitral valve *R*_*mva*_ resistances are also not influential. Since we do not have left heart data, the residuals do not include left heart quantities, and therefore we expect these to be non-influential. This finding agrees with previous studies [10,17,45] that fix the valve resistances. In general, the data available for model calibration will dictate which components of the model to include in the sensitivity analysis. Additional data that involve states not analysed here (e.g. left ventricle volume or tricuspid flow velocity) may affect which parameters are the most influential.

Both local and global analysis techniques are essential as they each highlight model features. Global sensitivities identify influential parameters over the physiological parameter range, while local sensitivities are evaluated at known values. Global sensitivity analysis sample parameters over the physiological range, but due to nonlinear model behaviour, this could include combinations that generate a non-physiological output. Yet, the local analysis only provides a snapshot of the sensitivities; again, since the model is nonlinear, the parameter influence may change if a parameter is changed, i.e. a parameter influential before optimization could be non-influential after optimization. For example (figure 4), the atrial timing parameter *τ*_*c*,*a*_ is less influential for patients 3 and 5 than for the other PH patients, and the left ventrcular maximal elastance *E*_*M*,*la*_ is less influential for patient 4. These results agree with Marquis *et al.* [17], where left ventricle elastance and systolic timing parameters varied across each nominal parameter set. Global sensitivity analysis cannot identify these discrepancies, as it integrates the sensitivity over the physiological parameter space.

While influential parameters are consistent between methods, individual parameters may have a different ranking. As shown in figure 4, the maximal atrial elastance *E*_*M,ra*_ is the second most influential parameter in the global analysis, whereas the local analysis ranks the parameter significantly lower. This can be attributed to interactions between the right atrial maximal and minimal elastance, *E*_*M*,*ra*_ and *E*_*m,ra*_, which account for the right atrial reservoir and pump function. Note that small changes in minimal elastance *E*_*m,ra*_ drastically affect maximum and minimum pressure values, while changes in maximal elastance *E*_*M,ra*_ only affect the model output when *E*_*M,ra*_ ≫ *E*_*m,ra*_. Though the ranking of *E*_*M*,*ra*_ differs, *E*_*m,ra*_ is always influential. These differences in parameter influence and model sensitivity highlight the importance of using multiple methods for parameter subset reduction.

Deficiencies in right atrial reservoir and contractile function are strong predictors of mortality in PH [46]. Systemic venous dynamics and tricuspid valve integrity dictate right atrial filling during ventricular diastole. In the model, the right atrial systolic and diastolic pressures are determined by minimum elastance *E*_*m,ra*_, which is always influential. The tricuspid valve resistance *R*_*tva*_ forms the interface for right atrial and ventricular interaction. Hence, this parameter influences the relationship between the two heart chambers throughout the cardiac cycle. The high sensitivity of right atrial predictions to these parameters mimics the current physiological understanding of altered right atrial function in PH [46].

Two of the three parameters characterized differently between the local and global methods are timing parameters dictating contraction and relaxation of the heart. The timing of heart contraction and relaxation are well approximated from dynamic pressure data. Hence, the uncertainty in these parameters (i.e. the bounds for global sensitivity sampling) is substantially smaller (±10–15%) than other model parameter uncertainty (±400%). This contributes to why the Sobol’ indices are smaller than the local analysis. Since our nominal timing parameter values are well informed, the local analysis is more relevant and used to determine timing parameter influence.

The final parameter with varying influence is the systemic venous resistance *R*_*sv*_. This parameter impacts central venous pressure and right atrial filling. This parameter is situated at the border between influential and non-influential. The parameter is essential to predict atrial dynamics and therefore included in the subset.

### 4.2. Parameter inference and subset selection

We fix non-influential parameters at their nominal values; however, this does not guarantee that the parameter subset is practically identifiable [15,45]. We combine SVD-QR subset selection and multi-start parameter inference to determine an identifiable parameter subset. SVD-QR methods reduce the number of parameters [37], and multi-start inference tests if solutions to the inverse problem are unique. For each patient, our results provide consistent parameter estimates across both residuals. Results reveal that the model with static data has 11 identifiable parameters, while the model with static and dynamic data has 14 identifiable parameters. An important observation is that the identifiable parameter subsets are subsets of each other, i.e. $\theta^{\;r_{1}}\subset \theta^{\;r_{2}}$. These results demonstrate that the patient-specific model is robust.

Our finding that sensitivity analysis alone is inadequate for determining identifiable parameters agrees with previous results. For example, Schiavazzi *et al.* [16] reported that sensitivity analyses do not guarantee unique parameter estimates. The authors use multi-start inference to interrogate parameter identifiability and reduce their parameter subset. We use a similar technique. A CoV cut-off of 10%, shown in figure 5, ensures that parameter estimates are robust to simulations with 20% uncertainty in initial guesses.

As shown in figure 6, identifiable parameters, including the pulmonary vascular resistance *R*_*p*_, the tricuspid valve resistance *R*_*tva*_, minimum right atrial elastance *E*_*m*,*ra*_ and the minimum ventricular elastances, *E*_*m*,*rv*_ and *E*_*m*,*lv*_, are elevated in PH. The parameters *R*_*p*_ and *R*_*tva*_ have the largest relative increase. Pulmonary vascular resistance (PVR) is a known biomarker of PH disease severity. It is elevated for both PAH and CTEPH patients [47,48]. The increase in minimum elastance in the right atrium and ventricle indicates chamber stiffening, as reported in PH [30]. An elevated end-diastolic elastance, *E*_*m*,*rv*_, is negatively correlated with right atrial reservoir, passive and active strain [30], suggesting that right atrial and ventricular function deteriorates during PH progression. We also observe a slight elevation in minimal left ventricle elastance, *E*_*m*,*lv*_. PAH and CTEPH directly affect right ventricular function, yet right ventricular dysfunction will affect the left ventricle in severe cases. E.g. impaired left ventricular function can be attributed to rightward septal bulging in severe PH and may suggest decompensated heart failure [49]. Both *R*_*p*_ and *E*_*m*,*rv*_ contribute to pulmonary circulation pressure but *R*_*p*_ has a more significant effect on pulmonary artery systolic and pulse pressure. Another important disease biomarker is pulmonary arterial compliance *C*_*pa*_, which is similar to arterial distensibility. Figure 6 shows a relative decrease in *C*_*pa*_ with PH, which reflects stiffening of the proximal pulmonary arteries due to constitutive changes (e.g. collagen accumulation) [50,51].

Several studies [44,48,50,52,53] have emphasized the inverse relationship between pulmonary resistance *R*_*p*_ and compliance *C*_*pa*_, often referred to as RC-time, *τ* = *R*_*p*_*C*_*pa*_. Tedford *et al.* [44] report an inverse-hyperbolic relationship from analysis of data from 1009 patients with PH and normal pulmonary capillary wedge pressure with best-fit curve *C*_*pa*_ = 0.564/(0.047 + *R*_*p*_) and RC time *τ* = 0.48 ± 0.17 (s). Similarly, the retrospective study by Assad *et al.* [52] found that the RC time is *τ* = 0.7 ± 0.34 in PAH patients (*n* = 593) with a best-fit curve *C*_*pa*_ = 0.775/(0.045 + *R*_*p*_). They also noted that the inverse-hyperbolic RC-time relationship is nearly identical for PAH and group 2 PH patients. Figure 7 shows this relationship from our patient cohort. The best-fit curve *C*_*pa*_ = 0.6518/(0.1105 + *R*_*p*_) and constant RC time *τ* = 0.55 ± 0.15 are consistent with results from these studies [44,52]. Our results were obtained from analysis of a closed-loop model, whereas the original RC times are computed using an isolated Windkesel model. This suggests that our systems-level model reproduces key features across large PH cohorts. Patients 1, 2, 3 and 5, located in the lower right portion of figure 7, have the greatest systolic right ventricular and main pulmonary aretery pressure. Prior studies [44] have also found this correlation between disease severity and RC-time.

The parameters in the static and dynamic residuals, including the systemic venous resistance controlling flow from the systemic veins to the right atrium, significantly affect right atrial filling. PH patients have a slight reduction in systemic venous resistance *R*_*sv*_ relative to the normotensive patient, increasing systemic venous inflow and diastolic right atrial filling. Growing evidence suggests that right atrial function is impaired during PH, though little is known about how the coupling of the right atrium and ventricle is altered with disease progression [2,46]. Using dynamic right atrium data for model calibration provide insight into the mechanisms of right atrial contractile and reservoir deterioration with right ventricular dysfunction. Changes in right atrial contractile timing can only be observed with dynamic pressure data. Other parameters only in the dynamic residual include ventricular and atrial timing parameters *T*_*c*,*v*_, *T*_*c*,*a*_ and *τ*_*c*,*a*_. These parameters are associated with the timing of heart function, i.e. the generation of the waveforms. Alenezi *et al.* [46] studied right atrial strain across 67 PAH subjects using speckle-tracking imaging. They found that right atrial dysfunction is an independent predictor of mortality and that the right atrial strain rate (time-dependent) correlates with PAH severity. Future investigations using right atrial pressure and strain data modelling may reveal additional indicators of right atrial dysfunction and PAH severity.

As shown in figure 8, including more data in the parameter inference procedure increases the number of identifiable parameters and changes model predictions. Both residuals account for systolic, diastolic and mean values, which are well matched by the model across all patients. Dynamic pulmonary artery and right ventricular predictions are unchanged between ***r***_1_ and ***r***_2_. This is attributed to good nominal estimates of the ventricular timing parameters *T*_*c*,*v*_ and *T*_*r*,*v*_, i.e. the optimized values are close to nominal values. By contrast, there is a significant change in simulated right atrial dynamics when calibrating the model to dynamic pressure data. The intricate dynamics of atrial filling and contraction make it challenging to visually identify the right atrial timing parameters from pressure data. The atrial pressure–volume loops in figure 8 vary significantly when comparing ***r***_1_ with ***r***_2_. The study by Domogo and Ottesen [43] studied left atrial dynamics using a systems-level model. Their model has a more sophisticated atrioventricular coupling, but the authors noted that an elastance model can capture dynamic atrial data. The time-varying dynamics of the atria are more complex, demonstrating the need for dynamic rather than static data for model calibration. The right atrium is gaining traction as a biomarker for PH severity [30,46]. Hence our ability to calibrate right atrial dynamics may provide further insight into the progression of right atrial–right ventricular–main pulmonary artery dysfunction in PH.

Since we do not have volume data, we included additional volume constraints in our inference procedure. It is well established that both PAH and CTEPH cause increased right ventircular myocardial remodelling, including wall thickening and dilatation [30]. Penalizing the inference procedure to ensure BSA-indexed blood volumes in all cardiac chambers constrains the model forecasts to volumes seen in clinical studies [30]. The addition of constraints leads to increased right atrial filling volumes and pressure magnitudes, as noted by Tello *et al.* [30]. Moreover, as shown in figure 9, the right ventricular pressure–volume loop has a rightward shift but is comparable in shape to its left ventricle counterpart. This shift is known to occur in PH [54], increasing the end-systolic elastance. Our results show a reduction in left ventricle stroke volume due to right ventricular dysfunction in several patients. A recent study by Jayasekera *et al.* [55] reported significant decreases in left ventricular strain and mechanical dyssynchrony in a cohort of PAH patients.

PH diagnosis uses RHC to determine right ventricle and pulmonary circulation pressures. However, these data alone provide little information on cardiac function or how the systemic circulation has adapted to disease. Simulation-derived outcomes provide these details, including stroke work, resistance, compliance ratios, and the pulsatility index. This study predicted cardiac stroke work, a known indicator of cardiac oxygen consumption and overall cardiomyocyte function. Clinically, stroke work is calculated as the product of stroke volume and mean arterial pressure; using the model, stroke work is calculated by determining the area of the pressure–volume loop. Both left and right ventricular stroke work, listed in table 6, change in PH. Left ventricular stroke work generally decreases, while right ventricular stroke work increases in PH. These findings agree with the retrospective clinical analysis by Chemla *et al.* [56], who found that right ventricular stroke work doubled in PH. Increased right ventricular stroke work is linked to severe paediatric PAH in a study by Yang *et al.* [23], who also use a compartment model to generate pressure–volume loops. Without volume data, our model can provide these indicators of disease severity, making them clinically relevant. Obtaining ventricular volumes during RHC would increase the number of identifiable parameters in the system. Several clinician-scientists advocate this [55,57], but it requires access to advanced imaging technologies. Given this disparity in access, computational models can provide additional simulated quantities without measurements.

### 4.3. Uncertainty quantification

We efficiently determined both parameter and output uncertainty using frequentist analyses. This study only infers identifiable parameters. More influential parameters have narrower confidence intervals compared with less influential ones (table 5). A consequence of narrow parameter bounds is that the model confidence and prediction intervals sensitive to these influential parameters contain the measured data remarkably well for both residuals.

Output uncertainty is compared in figure 10 for the two residuals ***r***_1_ or ***r***_2_. Model outputs computed using ***r***_1_ have relatively small uncertainty for static targets. For ***r***_2_, including both static and dynamic data, the uncertainty increases significantly, probably due to the increased complexity of the inverse problem. The least squares error is considerably higher, and even though the model does an excellent job fitting data, there are parts of the waveform that the simple lumped model used here cannot reproduce. However, we gain information about the dynamic output uncertainty in the dynamic right atrial, right ventriccular and main pulmonary artery predictions using ***r***_2_. This better quantifies the expected beat-to-beat variation we would expect to see on continuous RHC monitoring. In general, a more liberal estimate of uncertainty, as shown from ***r***_2_, reduces the chance of having a biased prediction due to a single heartbeat of data.

Other groups have performed uncertainty quantification on cardiovascular models. The study by Harrod *et al.* [45] investigated PA pressure uncertainty using Markov chain Monte Carlo sampling. Their study focuses on model outputs and parameter uncertainties using normotensive and PH data, similar to our analysis. Several authors have performed uncertainty quantification using one-dimensional [41,58] or three-dimensional [59] fluid dynamics models, which are fundamentally different from the systems-level model used here. Colebank *et al.* [41] found that uncertainty bounds around main pulmonary artery pressures were nearly identical between frequentist or Bayesian methods. The study also compared uncertainty across normotensive and hypoxia-induced PH mice. It revealed a larger uncertainty in the normotensive mice due to a more significant discrepancy in the model fit to data. Our results show a similar trend, with larger uncertainty typically attributed to patients with more complex RA dynamics (see electronic supplementary material). Our zero-dimensional model cannot capture the dynamics of wave reflections suitable for a zero-dimensional haemodynamics model. Yet, it does capture the global diastolic decay in pulmonary artery pressure, as shown in figure 10. We match the model to right ventricular dynamics exceptionally well; note the narrow confidence and prediction intervals in figure 10. The study by Yang *et al.* [23] captured right ventricular mechanics in PH using a simplified, open-loop model. We show that a more complex model accounting for the systemic circulation and left heart can still accurately predict right ventricular dynamics.

### 4.4. Limitations

This study has several limitations. Our model accounts for left and right ventricular dynamics without including interventricular interaction via the septal wall. Several studies have included this mechanism [49], which is essential for understanding how the right ventricle affects left ventricular function. Adding this model component provides a next step in understanding biventricular function during PH progression [55]. We use data from nine patients, four with CTEPH and five with PAH. We do not have a sufficiently large sample size to deduce differences in PH phenotypes, though recent studies have found differences in the biomechanics of the two subgroups [57]. However, we show that our zero-dimensional systems-level model can efficiently integrate multi-modal data and deduce markers of PH severity. Our inference procedure enforces cardiac volumes that match previously recorded BSA-indexed values; additional volume data (e.g. from a conductance catheter) would better inform the model calibration. Yet these were not available for all patients studied here.

Moreover, the data are measured using different technologies, probably with varying measurement errors. Another source of variability comes from physiological beat-to-beat variations in signals (e.g. due to respiration, circadian rhythms and anxiety). The model used here is relatively simple. Hence model mismatch provides another source of uncertainty. To prevent adding unwarranted bias, this study scaled residuals by the data generating residuals weighted to unity. We recognize that this choice of residual vector is problem specific. Therefore, any study using the methodology proposed here should assess this question in detail. However, the data used here are routinely collected during RHC and are consequently informative for future PH studies. As a result, studies using similar data and models can use the approach presented here, demonstrating that adding waveform data improves parameter inference and model calibration.

Parameter and output uncertainty is determined from asymptotic analyses, yet these rely on a Gaussian sampling distribution assumption. In our previous studies, Colunga *et al*. [9] and Marquis *et al.* [17] compared asymptotic predictions with Bayesian inference for a similar model. Results showed that the methods provide similar uncertainty estimates. The control patient was defined using literature pressure and volume measurements. Establishing a representative control cohort using sampling methodologies [45] probably would provide more robust comparisons between normotensive and PH model parameters. Lastly, it is well established that PH disproportionately affects women, with sex differences being a significant area of attention in the PH community [60]. Combining a larger, more diverse patient cohort in the parameter inference performed here may elucidate sex-dependent differences in right atrium, right ventricle and pulmonary artery, parameters. Our study is a proof of concept that patient-specific models can be constructed from RHC data, laying the foundation for future studies on a larger population of patients.

## 5. Conclusion

This study uses a zero-dimensional, systems-level haemodynamics model to predict changes in cardiovascular parameters in PH. We use sensitivity analyses and subset selection to deduce the best parameter subsets for two residuals: one with static data and one with additional dynamic right atrial, right ventricular and main pulmonary artery pressure waveforms. Our results show that adding time-series waveform data allows for additional parameters: systemic venous resistance *R*_*sv*_, the start of atrial contraction *τ*_*c*,*a*_, and peak ventricular contraction *T*_*c*,*v*_ to be estimated without altering estimates in the static-only residual. These additional parameters better describe the right atrial pump and reservoir function, which has been the focus of recent attention in the PH community [46]. Overall, model outcomes are consistent with the physiological understanding of the disease; PH induces increased pulmonary vascular resistance, decreased pulmonary arterial compliance, and elevated minimum right atrial and ventricular elastance, leading to increased mean pulmonary arterial pressure. While the uncertainty in model predictions is smaller for the static residual, adding time-series data provide valuable insight into uncertainty in dynamic predictions. Our study proves that systems-level models can be tuned to fit PH data. The model can predict the right atrial function by adding static and dynamic data, essential for differentiating PH subtypes. The framework devised here may be able to explain the mechanisms contributing to abnormal right atrial, right ventricular and main pulmonary artery function in PH.

## 6. Citation diversity statement

In agreement with the editorial from the Biomedical Engineering Society [61] on biases in citation practices, we have analysed the gender and race of our bibliography. This is done manually, though automatic probabilistic tools exist (e.g. https://zenodo.org/record/4104748#.YvVXpnbMI2z). We recognize existing race and gender biases in citation practices and promote the use of diversity statements encouraging fair gender and racial author inclusion.

Our references, including those in the electronic supplementary material, contain 15.15% woman (first)/woman (last), 13.64% man/woman, 16.67% woman/man and 54.55% man/man. This binary gender categorization is limited because it cannot account for intersex, non-binary or transgender people. In addition, our references contain 6.06% author of colour (first)/author of colour (last), 12.12% white author/author of colour, 18.18% author of colour/white author and 63.64% white author/white author. Our approach to gender and race categorization is limited in that gender and race are assigned by us based on publicly available information and online media. We look forward to future databases allowing all authors to self-identify race and gender in an appropriately, anonymized and searchable fashion and new research that enables and supports equitable practices in science.

## Data Availability

This study uses retrospective data available along with computer code at https://github.com/mjcolebank/CDG_NCSU/. The data are provided in electronic supplementary material [62].
